# Blood Lead Levels and Death from All Causes, Cardiovascular Disease, and Cancer: Results from the NHANES III Mortality Study

**DOI:** 10.1289/ehp.9123

**Published:** 2006-07-06

**Authors:** Susan E. Schober, Lisa B. Mirel, Barry I. Graubard, Debra J. Brody, Katherine M. Flegal

**Affiliations:** 1 Division of Health and Nutrition Examination Statistics, National Center for Health Statistics, Centers for Disease Control and Prevention, Hyattsville, Maryland, USA; 2 Harris Corporation, Falls Church, Virginia, USA; 3 Division of Cancer Epidemiology and Genetics, National Cancer Institute, Rockville, Maryland, USA

**Keywords:** cancer, cardiovascular disease, lead, mortality, National Health and Nutrition Examination Survey (NHANES), United States

## Abstract

**Background:**

Analyses of mortality data for participants examined in 1976–1980 in the second National Health and Nutrition Examination Survey (NHANES II) suggested an increased risk of mortality at blood lead levels > 20 μg/dL. Blood lead levels have decreased markedly since the late 1970s. In NHANES III, conducted during 1988–1994, few adults had levels > 20 μg/dL.

**Objective:**

Our objective in this study was to determine the risk of mortality in relation to lower blood lead levels observed for adult participants of NHANES III.

**Methods:**

We analyzed mortality information for 9,757 participants who had a blood lead measurement and who were ≥ 40 years of age at the baseline examination. Using blood lead levels categorized as < 5, 5 to < 10, and ≥ 10 μg/dL, we determined the relative risk of mortality from all causes, cancer, and cardiovascular disease through Cox proportional hazard regression analysis.

**Results:**

Using blood lead levels < 5 μg/dL as the referent, we determined that the relative risk of mortality from all causes was 1.24 [95% confidence interval (CI), 1.05–1.48] for those with blood levels of 5–9 μg/dL and 1.59 (95% CI, 1.28–1.98) for those with blood levels ≥ 10 μg/dL (*p* for trend < 0.001). The magnitude of risk was similar for deaths due to cardiovascular disease and cancer, and tests for trend were statistically significant (*p* < 0.01) for both causes of death.

**Conclusion:**

In a nationally representative sample of the U.S. population, blood lead levels as low as 5–9 μg/dL were associated with an increased risk of death from all causes, cardiovascular disease, and cancer.

Toxic effects of exposure to high levels of lead among adults in occupational settings and from poisonings include neurologic and renal impairment as well as effects on other organ systems [Agency for Toxic Substances and Disease Registry (ATSDR) 1999]. Environmental exposure to lead—exposure much lower than that found in occupational settings—is associated with long-term adverse effects, particularly on blood pressure (Cheng et al. 2001; Glenn et al. 2003; Hertz-Picciotto and Croft 1992; Hu et al. 1996; Moller and Kristensen 1992; Nash et al. 2003), renal function (Kim et al. 1996; Muntner et al. 2003; Payton et al. 1994; Staessen et al. 1992; Tsaih et al. 2004), and cognition (Payton et al. 1998; Weisskopf et al. 2004; Wright et al. 2003). Recent reports suggest that even blood lead levels that are currently typical in the U.S. population are associated with renal dysfunction (Muntner et al. 2005) and peripheral arterial disease (Muntner et al. 2005; Navas-Acien et al. 2004). Lead and lead compounds are reasonably anticipated to be human carcinogens based on evidence from occupational mortality and animal studies [National Toxicology Program (NTP) 2005].

Lead exposure declined dramatically beginning in the late 1970s largely because of mandated removal of lead from gasoline, from paint, and to a lesser extent, from solder used in cans (Annest et al. 1983; Pirkle et al. 1994). Blood lead measurements from the National Health and Nutrition Examination Surveys (NHANES) have provided documentation of lead exposure in the U.S. population beginning with data from NHANES II conducted during 1976–1980. Among person 1–74 years of age, the geometric mean of blood lead level dropped from 12.8 μg/dL in 1976–1980 to 2.8 μg/dL in 1988–1991 (Annest et al. 1983), and to 2.3 μg/dL in 1991–1994 [Centers for Disease Control and Prevention (CDC) 1997].

Analyses of mortality follow-up data for participants of NHANES II suggested an increased risk of mortality at blood lead levels > 20 μg/dL (Jemal et al. 2002; Lustberg and Silbergeld 2002). Mortality follow-up through the year 2000 is now available for NHANES III participants. The purpose of our study was to examine the risk of mortality from all causes, cardiovascular disease, and cancer in relation to blood lead levels observed for adult participants of NHANES III.

## Methods

NHANES III was fielded in two phases, the first from 1988 through October 1991 and the second from November 1991 through 1994. The survey consisted of a household interview and a standardized physical examination in a mobile examination center. The NHANES III sample was selected through a complex, multistage probability design. The survey has been described in detail elsewhere (CDC 1994).

During the physical examination, blood was obtained by venipuncture for all survey participants ≥ 1 year of age. Blood lead concentrations were measured by graphite furnace atomic absorption spectrophotometry and expressed in micrograms per deciliter. Details regarding laboratory methods and quality control procedures have been described previously (Pirkle et al. 1994).

NHANES III participants ≥ 17 years of age were eligible for passive mortality follow-up, which has been completed for deaths occurring through December 2000. Mortality information is based on the results of a probabilistic match between NHANES III and the National Death Index records [National Center for Health Statistics (NCHS) 2006].

This analysis was limited to persons ≥ 40 years of age at the time of the examination. Of the 10,202 persons ≥ 40 years of age, 9,762 (95.7%) had a blood lead measurement at baseline. Five persons were excluded because of insufficient information to allow for follow-up, resulting in a sample of 9,757 persons. The median length of follow-up was 8.55 years, during which there were 2,515 deaths (25.8% of participants). The *International Classification of Diseases, Tenth Revision* (ICD-10; NCHS 2006) was used to identify deaths due to malignant neoplasm (ICD-10 codes C00–C97) and major cardiovascular disease (ICD-10 codes I00–I78). Persons who were not matched to a death record were considered alive through the follow-up period and administratively censored 31 December 2000.

Blood lead levels were categorized as < 5, 5 to < 10, and ≥ 10 μg/dL. The sample size for persons with blood lead levels ≥ 20 μg/dL was too small to provide reliable estimates of mortality risk for this group. Because of the skewed distribution of blood lead concentrations, log-transformed values were used for additional analyses using lead levels as a continuous variable.

A participant’s age was defined as his or her age in years at the baseline examination. Race/ethnicity was categorized as non-Hispanic white, non-Hispanic black, Mexican American, and other. Education level was categorized as < 12 years or ≥ 12 years of education. Total family income was defined as < $20,000/year or ≥ $20,000/year. Region, as defined by the U.S. Census Bureau (2006), was recorded as Northeast, Midwest, West, or South. Urban status of residence was defined by population size and place of residence, and categorized into two groups: central or fringe counties of metropolitan areas with population size of ≥ 1 million versus all other areas. History of cigarette smoking was categorized as never, former, or current. Alcohol consumption was used as a continuous variable and defined by summing the number of drinks of beer, wine, or liquor consumed per week. More than 21 drinks was truncated and counted as 21.

We used Cox proportional hazard regression analysis, using age as the time scale to examine the relative hazard (or relative risk) of mortality from all causes, cancer, and cardiovascular disease using categories of blood lead concentrations as described above (Korn et al. 1997). As recommended by Korn et al. (1997), we stratified the baseline hazard by birth cohort; we used 6-year intervals. This controls for potential cohort differences in cumulative exposure to lead before the late 1970s, when current exposure to lead began to decline dramatically (Annest et al. 1983; Pirkle et al. 1994). Blood lead levels largely reflect current exposure (Rabinowitz et al. 1976) and are an imperfect measure of a person’s lifetime exposure to lead. Because current lead exposure and blood lead levels continued to decline over the 6-year period of blood collection in NHANES (CDC 1997; Pirkle et al. 1994), we also stratified the baseline hazard by survey phase. For cause-specific analyses, follow-up for those who died from other causes was censored at the age of death.

Initial models assessed univariate associations of covariates and mortality. Next, we constructed multivariate proportional hazard models to examine the association of lead exposure and mortality after adjusting for potential confounders. All two-way interactions with blood lead category were assessed. We assessed the proportional hazard assumption by examining the consistency of the relative hazards of mortality across three age groups. We defined three age categories, 40–74, 75–84, and ≥ 85 years, so that there were an approximately equal number of deaths from all causes in each category. Study subjects can be included in more than one age group as their ages change during the follow-up period. Because the cancer mortality and blood lead relationship was different for men and women in a previous study of the NHANES II cohort, we also stratified multivariate models separately for males and females.

We analyzed the dose–response relationship of blood lead and mortality in two ways. First, the multivariate-adjusted relative risks across the three blood lead categories were tested for trend. A linear term consisting of the median values for each lead group was placed in the proportional hazards model instead of the dummy variables for each group, and this linear term was analyzed using a Wald test. Second, the dose–response relationship between lead and mortality from all causes was evaluated using log-transformed blood lead concentration as a continuous variable with a five-knot cubic regression spline in the multivariate proportional hazards model (Durrleman and Simon 1989). We used a Wald test to evaluate the dose–response relationship.

Statistical analyses were conducted using the SAS System for Windows (release 9.1; SAS Institute Inc., Cary, NC) and SUDAAN (release 9.0; Research Triangle Institute, Research Triangle Park, NC). All analyses included sample weights that accounted for the unequal probabilities of selection and nonresponse. All variance calculations incorporated the sample weights and accounted for the complex sample design using Taylor series linearization. All significance tests were two-sided using *p* < 0.05 as the level of statistical significance.

## Results

Table 1 summarizes characteristics of the NHANES III cohort by blood lead categories. Person-years of follow up were approximately 53,000, 20,000, and 4,500 by blood lead categories < 5, 5–9, and ≥ 10 μg/dL, respectively. Remaining estimates are weighted by the sample weights. Blood lead levels increased with age. Blood lead concentrations were disproportionately higher for men, non-Hispanic blacks, those with low income, those with educational attainment of < 12 years, current smokers, and residents of the Northeast, and persons who participated in the first phase of the survey. Alcohol intake was positively associated with blood lead concentrations. No differences in blood lead concentrations by urban status of residences were noted.

The number of deaths and multivariate-adjusted relative risks of mortality due to all causes, cardiovascular disease, and cancer by blood lead category are presented in Table 2. The multivariate results exclude 71 persons with missing information on education level, for a final sample size of 9,686. After this exclusion, there were a total of 2,485 deaths from all causes, 1,189 from cardiovascular disease, and 543 from cancer. For each category of deaths, statistical testing did not support the null hypothesis that proportional hazards were constant by age, although the lead-mortality relationships were generally consistent across age strata. For completeness, age-stratified results are shown in addition to results for all ages combined. The following covariates were included in all multivariate models: sex, race/ethnicity, education level, and smoking status. Census region and urban status of residence were not predictive, and alcohol intake was weakly predictive of mortality in our analysis. Including these three covariates in the analysis did not alter the blood lead and mortality relationships; therefore, we excluded these three covariates from the final multivariate models. Socioeconomic status, as indicated by either annual family income (categorized as < $20,000 and ≥ $20,000) or education level was an important confounder in the multivariate analyses. We chose to use education level rather than income in our final models because there was less missing information for this variable. In general, interaction terms between lead and covariates were not statistically significant. We found a *p*-value of 0.02 for the interaction terms lead × education in the cardiovascular disease model and lead × race/ethnicity in the cancer mortality model; however, the interactive effects were not large and did not alter the direction of the lead–mortality relationships. Therefore, we did not include these terms in our final models.

In general, we found an increased risk of mortality from all causes with increasing blood lead levels (Table 2), where the reference category was blood lead levels < 5 μg/dL. For all ages combined, the relative risks of mortality were 1.24 [95% confidence interval (CI), 1.05–1.48) for those with blood levels of 5–9 μg/dL and 1.59 (95% CI, 1.28–1.98) for those with blood levels of ≥ 10 μg/dL (test for trend, *p* < 0.001). For mortality due to cardiovascular disease, there was also a pattern of increasing risk with increasing blood lead. For all ages combined, the estimated relative risk of mortality from cardiovascular disease was 1.20 (95% CI, 0.93–1.55) for those with blood lead levels of 5–9 μg/dL and 1.55 (95% CI, 1.16–2.07) for those with blood lead levels of ≥ 10 μg/dL (test for trend, *p* < 0.01). For all ages combined, the estimated relative risk of mortality from cancer was 1.44 (95% CI, 1.12–1.86) for those with blood lead levels of 5–9 μg/dL and 1.69 (95% CI, 1.14–2.52) for those with blood lead levels of ≥ 10 μg/dL (test for trend, *p* < 0.01).

**Figure 1:**
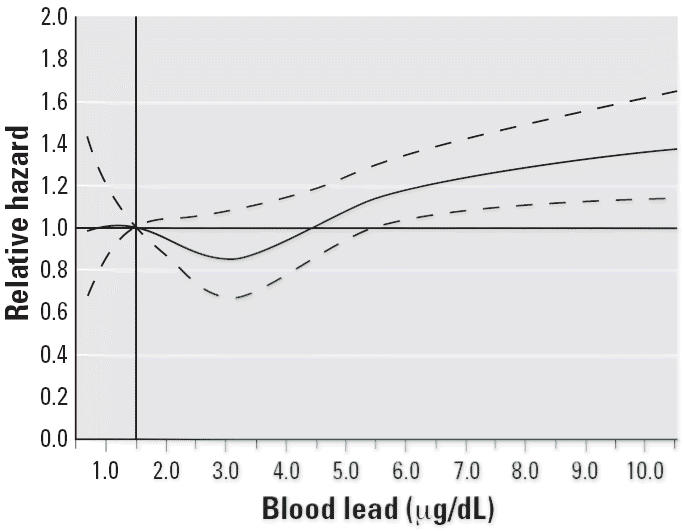
Relative risk of all cause mortality for different blood lead levels compared with referent level of 1.5 μg/dL (12.5th percentile). The solid line shows the fitted five-knot spline relationship; the dashed lines are the point-wise upper and lower 95% CIs.

We further explored the dose–response relationship between blood lead concentrations and mortality from all causes by modeling the proportional hazards using log-transformed blood lead levels on a continuous scale with a five-knot cubic regression spline. The spline analysis results displayed as relative hazards using a referent blood lead level of 1.5 μg/dL (12.5th percentile) and 95% CIs are shown graphically in Figure 1. This result is adjusted for all covariates included in the final model shown in Table 2. The lower bound of the 95% CI of the relative hazard exceeds unity at a blood lead concentration of approximately 5.5 μg/dL. The relative hazard remains < 2 for blood lead levels ≤ 10 μg/dL.

## Discussion

In this analysis of mortality among a large cohort of adults representative of the U.S. population, we found an increased risk of death from all causes, cardiovascular disease, and cancer associated with elevated blood lead levels. Overall, the increase in risk was small; however, the increase was observed for blood lead levels as low as 5–9 μg/dL. Our finding of dose–response relationships between mortality risk and increasing blood lead levels for all three classes of death strengthens the conclusion that blood lead levels are associated with an increased risk of mortality.

Previous research has examined mortality risk and blood lead levels in the general population of the United States using mortality follow-up data for participants 30–74 years of age from NHANES II, a study with baseline data collected during 1976–1980 and follow-up through 1992 (Jemal et al. 2002; Lustberg and Silbergeld 2002). Lustberg and Silbergeld (2002) analyzed data for persons of all races and concluded that risk of mortality from all causes, circulatory disease, and cancer was elevated for persons with blood lead levels of 20–29 μg/dL compared with those with levels < 10 μg/dL. Multivariate-adjusted results did not show an increased risk for those with blood lead levels of 10–19 μg/dL. In an analysis limited to white participants, Jemal et al. (2002) found no dose–response relationship between quartiles of blood lead and risk of death from cancer, but they did find evidence of a threshold effect for women, with risk becoming significantly elevated at a blood lead concentration of approximately 24 μg/dL.

The results of our study showing increased mortality at levels as low as 5–9 μg/dL are consistent with other research that suggests health effects associated with low levels of lead exposure. Recent cross-sectional analyses of the current, ongoing NHANES, with data from 1999–2002, suggest an increased risk of peripheral arterial disease, hypertension, and renal dysfunction in a population with blood lead levels of approximately 2 μg/dL on average (Muntner et al. 2005; Navas-Acien et al. 2004). Other analyses of population-based studies support these results. For example, in the Normative Aging Study, blood lead levels < 10 μg/dL were associated with renal function and cognitive impairment (Kim et al. 1996; Payton et al. 1994, 1998; Weisskopf et al. 2004; Wright et al. 2003). Cross-sectional analyses of the NHANES III cohort have shown that blood lead levels are related to increased blood pressure (Nash et al. 2003) and decreased renal function (Muntner et al. 2003).

Exposure misclassification is a potentially important limitation of our analysis. We classified exposure based on blood lead levels measured at only one point in time. Most (75–95%) lead in the body is found in bone (Saltzman et al. 1990), where the half-life is 5–19 years (Rabinowitz 1991). Therefore, measures of bone lead indicate cumulative, long-term exposure and are the best measure to study chronic effects. The half-life of lead in blood is approximately 1 month (Rabinowitz et al. 1976); thus blood levels are related to current exposure. Lead is released from bone through resorption, such as occurs with aging, and this contributes to levels found in blood (Hu et al. 1998; Smith et al. 1996). Older persons in the NHANES III cohort were more likely to have higher cumulative lead exposure, and their blood lead levels may be disproportionately influenced by release of lead from bone stores compared with younger persons.

That our results show an increased risk of mortality at lower blood lead levels than the mortality studies of the NHANES II cohort may be related to exposure classification being based on a single blood lead measurement. Blood lead levels of NHANES III participants were close to 80% lower than in the NHANES II cohort, and few adults had blood lead levels > 20 μg/dL (CDC 1997; Pirkle et al. 1994). This reflects reductions in the current exposure of the study participants because of the elimination of lead from gasoline and other environmental sources. We speculate that the difference in cumulative lead exposure among adults across the two surveys is relatively smaller than the difference in current exposure. For example, it is likely that within the same birth cohort, most persons who had blood lead levels > 20 μg/dL in 1976–1980 would have levels < 10 μg/dL in 1988–1994. The particularly dramatic decrease in blood levels that occurred during the NHANES II (Annest et al. 1983) makes the evaluation of a dose–response relationship of blood lead level and mortality even more problematic and may explain why the previous analyses of NHANES II (Jemal et al. 2002; Lustberg and Silbergeld 2002) did not find as strong a dose effect as in our present analyses of NHANES III, which had more stable blood lead levels.

It is not possible to differentiate an acute effect of lead in blood from a chronic effect in studies that only use blood lead levels to indicate exposure (Hu et al. 1998). With advances in measuring bone lead through X-ray fluorescence, several studies have evaluated the association of health effects with both bone and blood lead measures. Both cross-sectional and longitudinal analyses of the Normative Aging Study suggest that cumulative exposure, as well as current exposure, to lead is associated with declines in renal function (Kim et al. 1996; Payton et al. 1994) and cognition (Payton et al. 1998; Weisskopf et al. 2004; Wright et al. 2003). Results from the Normative Aging Study suggest that cumulative but not current exposure is associated with an increased risk of hypertension (Tsaih et al. 2004); however, in a longitudinal study of a cohort who had previous occupational exposure, Glenn et al. (2003) concluded that the effect of lead on blood pressure is consistent with both acute and chronic modes of action.

From the present study we conclude that mortality is associated with lead exposure as indicated by a single blood lead measurement typical for the U.S. adult population during 1988–1994. Extrapolating from this to mortality risk related to cumulative lead exposure or blood lead measurements from different calendar periods can only be speculative. The blood lead and mortality relationship that we observed may be due to lead being a surrogate for some other factor that is associated with an increase in mortality. For example, socioeconomic status may not be sufficiently controlled for by measures of education level or family income. However, we adjusted for well-know confounders without observing a substantial reduction in the blood lead relationship. Also, the observed relationships for each category of mortality were fairly consistent across age and groups.

The major strengths of our study, that the NHANES cohort is representative of the U.S. population and that the sample size is large enough to evaluate small differences in risk, are notable. Our analysis adds to the body of evidence demonstrating adverse health consequences related to blood lead levels that fall below current levels of concern. Further research is needed to assess cumulative exposure and adverse health consequences, including mortality, for persons who were not exposed to high levels of lead in the environment that were typical before lead was removed from gasoline in the late 1970s.

## Figures and Tables

**Table 1:** Characteristics of the study cohort by blood lead level.

	Blood lead level (μg/dL)		
Characteristic	< 5	5–9	> 10
Sample size	6,608	2,532	617
Person-years	53,398	19,939	4,509
Median blood lead level (μg/dL)	2.6	6.3	11.8
Mean age at baseline (years)	57.0	60.6	62.0
Male (%)	39.1	65.2	74.2
Race/ethnicity (%)			
Non-Hispanic white	82.7	78.1	65.7
Non-Hispanic black	7.4	12.3	22.6
Mexican-American	3.4	3.5	4.3
Other	6.5	6.1	7.5
Total family income < $20,000 (%)	29.8	41.2	52.3
Education level < 12 years (%)	26.2	39.0	50.8
Smoking status at baseline (%)			
Never	48.1	26.1	19.3
Former	33.8	39.0	36.5
Current	18.1	34.9	44.2
Mean alcohol intake (drinks/week)	2.0	4.6	5.5
Census region (%)			
Northeast	17.4	29.1	36.2
Midwest	25.1	25.2	17.8
South	36.3	27.8	31.7
West	21.2	18.0	14.3
Urban status (%)	45.6	52.1	51.3
Phase 1 of survey (%)	44.4	58.2	65.3

**Table 2:** Multivariate adjusted^a^ relative risks for all-cause, cancer, and cardiovascular disease mortality by blood lead level and age category.

		Relative risk (95% CI) by age category (years)			
Cause of death/blood lead level	No. of deaths	40–74	75–84	> 85	All
All causes					
< 5 μg/dL	1,402	1	1	1	1
5–9 μg/dL	828	1.30 (1.03–1.65)	1.38 (1.04–1.83)	0.98 (0.85–1.14)	1.24 (1.05–1.48)
≥ 10 μg/dL	255	1.73 (1.28–2.35)^b^	1.39 (0.93–2.08)^c^	1.67 (1.11–2.53)	1.59 (1.28–1.98)^b^
Cardiovascular disease					
< 5 μg/dL	684	1	1	1	1
5–9 μg/dL	394	1.11 (0.79–1.56)	1.41 (0.87–2.28)	1.07 (0.87–1.31)	1.20 (0.93–1.55)
≥ 10 μg/dL	111	1.47 (0.93–2.33)	1.71 (0.94–3.09)^c^	1.45 (0.85–2.48)	1.55 (1.16–2.07)^d^
Cancer					
< 5 μg/dL	282	1	1	1	1
5–9 μg/dL	194	1.44 (0.91–2.28)	1.46 (1.03–2.07)	1.44 (0.92–2.26)	1.44 (1.12–1.86)
≥ 10 μg/dL	67	2.27 (1.38–3.74)^d^	0.80 (0.38–1.69)	2.2 (1.13–4.29)^d^	1.69 (1.14–2.52)^d^

CI, confidence interval.

a Adjusted for sex, race/ethnicity, education, and smoking status.

b *p*-Value for trend test < 0.001.

c *p*-Value for trend test < 0.05.

d *p*-Value for trend test < 0.01.
